# Serum 4-hydroxynonenal associates with the recurrence of patients with primary cerebral infarction

**DOI:** 10.3389/fncel.2022.998512

**Published:** 2022-11-10

**Authors:** Xingliang Liu, Meiling Bai, Lei Fan, Zhan Lou

**Affiliations:** ^1^Department of Neurology, The First Affiliated Hospital of Hebei North University, Zhangjiakou, China; ^2^Hebei North University, Zhangjiakou, China

**Keywords:** 4-hydroxynonenal, recurrence, cerebral infarction, risk, patients

## Abstract

**Background:**

4-Hydroxynonenal (4-HNE), an α, β-unsaturated hydroxyalkenal, has been found to be associated with aspirin resistance, which is a risk factor for recurrent cerebral infarction. However, its effect on recurrent cerebral infarction is less defined. We designed this study to investigate the association between 4-HNE and increased risk of recurrent cerebral infarction.

**Methods:**

We recruited 189 patients with primary cerebral infarction from 2017 to 2019. According to the recurrence of cerebral infarction during the 3-year follow-up period, they were divided into two groups, namely, the non-recurrence group (*n* = 93) and the recurrence group (*n* = 96). All patients were analyzed to explore the risk factors for the recurrence of primary cerebral infarction and the predictive value of serum 4-HNE for the recurrence of cerebral infarction.

**Results:**

The levels of serum 4-HNE in patients of the recurrence group were significantly higher than that in patients of the non-recurrence group. There was a positive correlation between serum 4-HNE levels and the serum levels of triglyceride (*r* = 0.448, *p* = 0.008) and low-density lipoprotein cholesterol (LDL-C; *r* = 0.442, *p* = 0.002) in primary cerebral infarction patients. Cox proportional hazards modeling showed that demographic and certain clinical parameters, such as age, serum triglyceride levels, the National Institutes of Health Stroke Scale (NIHSS) scores, and serum 4-HNE levels, were independent factors for the recurrence in patients. The results of the receiver operating characteristic (ROC) curve showed that the area under the curve (AUC) value of serum 4-HNE in patients with cerebral infarction recurrence was 0.703, and when the cutoff value of serum 4-HNE was set at 42.34 ng/ml, the sensitivity and specificity values of serum 4-HNE in predicting recurrent cerebral infarction were 79.20 and 52.70%, respectively.

**Conclusion:**

Serum 4-HNE is an independent risk factor for the recurrence of patients with primary cerebral infarction, and it may become a new intervention way to prevent the recurrence of patients with cerebral infarction.

## Introduction

Cerebral infarction, also known as ischemic stroke, is one of the most common cerebrovascular diseases, accounting for about 70% of all acute cerebrovascular diseases (Zhao et al., 2022). In China, cerebral infarction has the characteristics of high morbidity, high recurrence rate, high disability rate, and high mortality rate (Liu et al., 2021). In addition, the recurrence rate of patients with cerebral infarction can be as high as 14–17%, accounting for 30% of new patients with stroke in China every year (Huang et al., 2021; Liu et al., 2021). At present, the specific molecular mechanism of cerebral infarction recurrence has not been revealed, but a large number of studies have analyzed the risk factors, such as age, low-density lipoprotein cholesterol (LDL-C), hypertension, white matter lesions, and heart disease, affecting the recurrence of cerebral infarction (Anniwaer et al., 2019). These risk factors are of great significance to effectively prevent and control the recurrence of cerebral infarction.

The underlying pathology of cerebral infarction is one of oxidative stress (Fan et al., 2019). Exogenous and endogenous oxygen radicals trigger lipid peroxidation by attacking polyunsaturated fatty acids in phospholipids of biological membranes and generate a complex series of products and new free radicals, among which aldehyde products are the main breakdown products and end products of lipid peroxidation reactions (Zheng et al., 2020; Wang et al., 2022). When compared with free radicals, aldehyde-based products are more stable and can diffuse to many cellular components and extracellular with the damaging potential of the original free radicals (Hou et al., 2020; Pozdnyakov et al., 2020). Aldehyde products can also react with some nucleophilic substances, such as thiol compounds, DNA, proteins, and phospholipids, interfering with the normal functional activities of cells, damaging cellular components, and leading to the development of diseases (Hou et al., 2020; Pozdnyakov et al., 2020). In addition, aldehyde-based products can also act as bioactive molecules that are involved in functional activities, such as cell signaling, cell proliferation, and gene expression, at very low non-toxic concentrations (Li et al., 2015). In conclusion, aldehyde-based products play an important role in the occurrence and development of many diseases.

4-Hydroxynonenal (4-HNE) is the most representative substance among the aldehyde products of lipid peroxidation, mainly produced by linoleic acid and arachidonic acid, etc., in lipid peroxidation (Castro et al., 2017; Gallo et al., 2020). Importantly, Guo et al. (2020) found that 4-HNE was closely related to aspirin sensitivity in patients with acute cerebral infarction, with higher levels associated with a greater risk of aspirin resistance in patients. Moreover, aspirin resistance is considered to be a risk factor for the recurrence of cerebral infarction (Yi et al., 2013; Wiśniewski et al., 2020), so 4-HNE may also be associated with the recurrence of cerebral infarction, but there is a lack of direct research evidence. In the present study, we compared the serum 4-HNE levels at the initial diagnosis of cerebral infarction in different patients with cerebral infarction and studied the effect of serum 4-HNE levels on the recurrence of cerebral infarction in patients with primary cerebral infarction.

## Patients and methods

### Patients

From January 2017 to June 2019, we prospectively recruited 189 patients with primary cerebral infarction. All patients or their guardians were informed about all aspects of this study and signed informed consent. In addition, the research protocol for this study was reviewed and approved by Ethics Committee of our hospital. Inclusion criteria were as follows: (1) age over 18 years old; (2) the first diagnosis of cerebral infarction; (3) CT and/or magnetic resonance imaging (MRI) confirmed evident focal neurological symptoms/signs; (4) complete clinical data and signed informed consent; and (5) all patients are sensitive to aspirin and can be treated with aspirin. Exclusion criteria were as follows: (1) cerebral hemorrhage or non-primary cerebral infarction, such as traumatic cerebral infarction or old cerebral infarction; (2) history of traumatic brain injury and cerebrovascular disease in the past 3 months; (3) history of antiplatelet, anticoagulant, or non-steroidal anti-inflammatory drugs (NSAIDs) medication; (4) malignant tumor, infectious disease, autoimmune disease, or organ dysfunction; (5) liver injury, kidney injury, chronic obstructive pulmonary disease, pneumonia, and other diseases affecting serum 4-hydroxytonic; and (6) glucocorticoid, low molecular weight heparin sodium, erythromycin, Salvia miltiorrhiza injection, Shuxuening injection, montelukast sodium, and other drugs that affect serum 4-hydroxytonic.

### Data collection

We collected the clinical data of patients at admission, such as age, gender, admission time, medication history, medical history, hospitalization history, laboratory test data, comorbidities (hypertension, diabetes, and coronary disease), and living habits (smoking). The National Institutes of Health Stroke Scale (NIHSS) was used to assess the severity of stroke in patients with cerebral infarction at the time of admission.

Criteria for recurrence of cerebral infarction were as follows: the symptoms of the patient worsened before or other new symptoms of cerebral infarction occurred and new lesions of cerebral infarction were found through transcranial brain CT or magnetic resonance imaging.

### Serological tests

Fasting peripheral blood was collected from all patients the next morning after admission and centrifuged (1,000 × *g*, room temperature, 10 min) to collect serum. We used an automatic biochemical analyzer (BS-280, Mindray) to detect levels of serum LDL-C, high-density lipoprotein cholesterol (HDL-C), triglyceride, and total cholesterol. In addition, we used Human 4-HNE ELISA Kit (CSB-E16214h, Cusabio Biotech) to detect the levels of 4-HNE.

### Follow-up protocol

All patients were followed up for 3 years after the first diagnosis of cerebral infarction. During the follow-up period, the patients were interviewed by telephone every 3 months to collect information on the recurrence of cerebral infarction. For the patients with regular review, only the information on the recurrence of cerebral infarction was collected through outpatient information.

### Statistical analysis

Data in the present study were analyzed by SPSS 19.0 software (SPSS Inc., Chicago, IL, USA). Qualitative data were presented as counts (%), and *p*-values were calculated using chi-square or Fisher’s exact test as appropriate. The Kolmogorov-Smirnov test was used to check whether quantitative data conformed to a normal distribution, data that conformed to a normal distribution were presented as [mean ± standard deviation (SD)], and unpaired Student’s *t*-test was used to compare differences and calculate *p*-values. Quantitative data that did not conform to a normal distribution were presented as the median [interquartile range (IQR)], and Mann-Whitney U-test was used to compare differences and calculate *p*-values. Spearman’s correlation coefficient was used to analyze the association of serum 4-HNE levels with other clinical features. Receiver operating characteristic (ROC) curves were constructed and the area under the curve (AUC) was calculated to assess the performance of serum 4-HNE levels in distinguishing between primary cerebral infarction patients with and without recurrence at 3 years after cerebral infarction.

## Results

### Recurrence of cerebral infarction and baseline data

We prospectively enrolled 202 patients with primary cerebral infarction in this study. However, during the 3-year follow-up period, 7 patients were excluded due to missing or incomplete follow-up data and 6 patients died due to other diseases. At last, a total of 189 patients were finally included in this study. During the 3-year follow-up period, 96 of 189 patients with primary cerebral infarction developed recurrent cerebral infarction, namely, 93 patients in the non-recurrence group and 96 in the recurrence group. In comparison of baseline data between these two groups we found that there is no significantly different between the two groups on gender, smoking, coronary disease, serum HDL-C, total cholesterol, and NIHSS scores, while the age, proportion of patients with hypertension and diabetes, the serum level of triglyceride, and LDL-C in the non-recurrence group are all significantly lower than those in the recurrence group (Table 1).

**TABLE 1:** Baseline data of primary cerebral infarction patients.

Variable	No-recurrence (*n* = 93)	Recurrence (*n* = 96)	t/χ^2^	*P*
Age (years)	63.25 ± 5.68	68.82 ± 6.39	4.281	0.028
Gender [male, *n* (%)]	52 (55.92%)	56 (58.33%)	0.113	0.737
Smoking [*n* (%)]	38 (40.86%)	40 (41.67%)	0.013	0.910
Hypertension [*n* (%)]	55 (59.14%)	79 (82.29)	12.272	< 0.001
Diabetes [*n* (%)]	18 (19.35%)	42 (43.75%)	12.970	< 0.001
Coronary disease [n (%)]	10 (10.75)	13 (13.54)	0.344	0.558
Triglyceride (mmol/L)	1.49 ± 0.18	1.87 ± 0.30	10.640	< 0.001
LDL-C (mmol/L)	3.01 ± 0.40	3.37 ± 0.49	5.503	< 0.001
HDL-C (mmol/L)	1.01 ± 0.21	1.05 ± 0.23	0.532	0.419
Total cholesterol (mmol/L)	4.28 ± 1.28	4.28 ± 1.17	0.089	0.823
NHISS scores	6.57 ± 1.42	6.82 ± 1.49	0.452	0.627

### Serum 4-hydroxynonenal in different cerebral infarction patients

**FIGURE 1:**
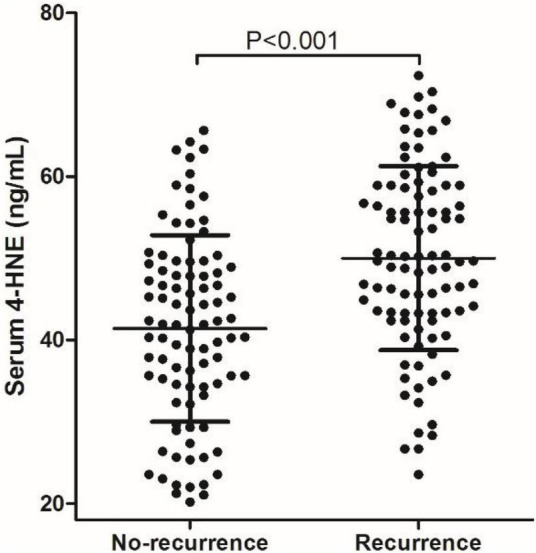
Comparison of serum 4-hydroxynonenal (4-HNE) level between non-recurrence group and recurrence group.

The mean value of serum 4-HNE level is 45.78 ng/ml (20.18–72.35 ng/ml) in 189 patients with primary cerebral infarction, 41.42 ng/ml (20.18–65.62 ng/ml) in 93 patients with no-recurrent primary cerebral infarction, and 50.00 ng/ml (23.56–72.35 ng/ml) in 96 patients with recurrent primary cerebral infarction. The mean value of serum 4-HNE was 41.42 ng/ml (20.18–65.62 ng/ml) in the non-recurrence group and 50.00 ng/ml (23.56–72.35 ng/ml) in the recurrence group. Importantly, the levels of serum 4-HNE in the recurrent group were significantly higher than that in the non-recurrence group (*p* < 0.05; Figure 1).

### Correlation between serum 4-hydroxynonenal and clinical data of cerebral infarction

**FIGURE 2:**
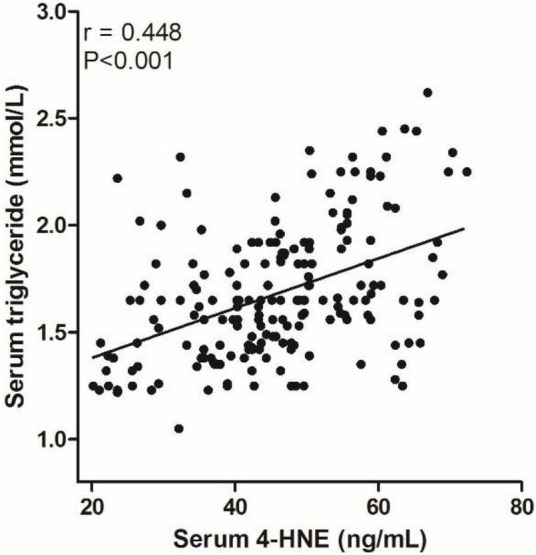
Scatter plot suggests a positive correlation between serum 4-hydroxynonenal (4-HNE) and triglyceride in primary cerebral infarction patients.

**FIGURE 3:**
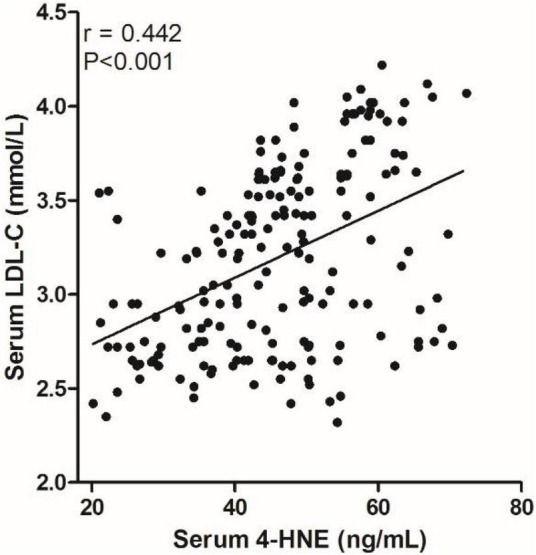
Scatter plot suggests a positive correlation between serum 4-hydroxynonenal (4-HNE) and low-density lipoprotein cholesterol (LDL–C) in primary cerebral infarction patients.

We analyzed the correlation between serum 4-HNE levels and clinical data of primary cerebral infarction patients and found that there is no significant correlation between serum 4-HNE levels and the age, gender, smoking, hypertension, diabetes, coronary disease, HDL-C, total cholesterol, and NIHSS scores of primary cerebral infarction patients, while there was a positive correlation between serum 4-HNE levels and the serum levels of triglyceride (Figure 2) and LDL-C (Figure 3) in primary cerebral infarction patients (Table 2).

**TABLE 2:** Correlation between serum 4-hydroxynonenal (4-HNE) and other variables using spearman correlation coefficient in primary cerebral infarction patients.

Variables	*R*	*P* value
Age	0.042	0.722
Gender	0.092	0.667
Smoking	0.035	0.828
Hypertension	0.209	0.083
Diabetes	0.108	0.304
Coronary disease	0.143	0.195
Triglyceride	0.448	0.008
LDL-C	0.442	0.002
HDL-C	0.095	0.663
Total cholesterol	0.038	0.809
NIHSS scores	0.038	0.811

### Predictive factors for recurrence of cerebral infarction

Univariate analysis showed that there were statistical differences in age, history of hypertension, triglyceride, low-density lipoprotein, NIHSS score, and 4-HNE level between the two groups. To further confirm whether 4-HNE was an independent factor, multivariate analysis was further performed, and the results showed that demographic and certain clinical parameters, such as age [hazard ratio (HR) = 1.071, 95% CI: 1.002–1.138, *p* < 0.001], serum triglyceride levels (HR = 1.628, 95% CI: 1.013–2.267, *p* = 0.042), NIHSS scores (HR = 1.421, 95% CI: 1.056–2.934, *p* = 0.023), and serum 4-HNE levels (HR = 2.631, 95% CI: 1.639–4.413, *p* < 0.001), were independent factors for recurrence in patients (Table 3).

**TABLE 3:** Multivariate Cox regression results of influencing factors for recurrent cerebral infarction.

Variables	Univariate analysis			Multivariate analysis		
	HR	95%CI	*P*	HR	95%CI	*P*
Age	1.121	1.032–1.321	< 0.001	1.071	1.002–1.138	< 0.001
Gender	0.932	0.634–1.567	0.925			
Smoking	0.689	0.441–1.328	0.192			
Hypertension	1.933	1.689–2.651	0.039	1.705	1.068–3.231	0.071
Diabetes	0.966	0.827–1.634	0.272			
Coronary disease	0.905	0.213–3.227	0.728			
Triglyceride	1.742	1.512–1.768	0.008	1.628	1.013–2.267	0.042
LDL-C	1.352	0.923–1.984	0.042	1.065	0.937–2.624	0.089
HDL-C	0.652	0.569–0.986	0.139			
Total cholesterol	0.936	0.892–1.657	0.413			
NIHSS scores	1.321	1.218–2.392	0.011	1.421	1.056–2.934	0.023
4-HNE	2.325	2.025–4.963	< 0.001	2.631	1.639–4.413	< 0.001

### Predictive value of serum 4-HNE on recurrent cerebral infarction

**FIGURE 4:**
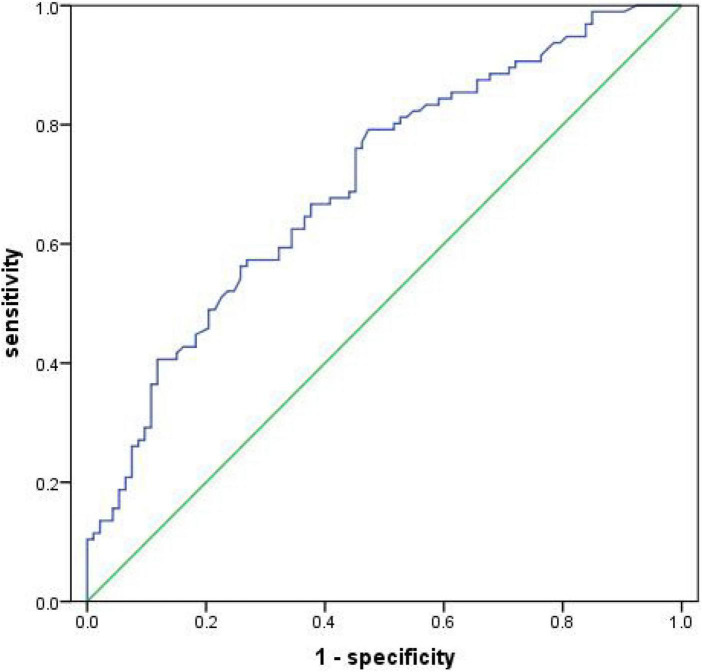
Receiver operator characteristic curve demonstrating a predictive value of serum 4-hydroxynonenal (4-HNE) on recurrent cerebral infarction.

The receiver operator characteristic curve was used to assess the predictive value of serum 4-HNE on recurrent cerebral infarction. The results showed that the AUC value of serum 4-HNE in patients with cerebral infarction recurrence was 0.703 (95% CI: 0.630–0.777, *p* < 0.001; Figure 4). In addition, when the cutoff value of serum 4-HNE was set at 42.34 ng/ml, the sensitivity and specificity values of serum 4-HNE in predicting recurrent cerebral infarction were 79.20 and 52.70%, respectively.

## Discussion

4-Hydroxynonenal is a biomarker of oxidative stress and plays an important role in the pathophysiology of diseases, such as ischemic stroke (Lee et al., 2012; Li et al., 2018), metabolic disease (Castro et al., 2017), coronary heart disease (Chapple et al., 2013; Rosen et al., 2018), and cancer (Zhong and Yin, 2015; Gasparovic et al., 2017). In a study on patients with cerebral infarction, the authors found that serum 4-HNEs were significantly higher in patients with cerebral infarction than in healthy subjects and that serum 4-HNE levels were associated with the severity of brain injury in patients, suggesting that serum 4-HNE is a biomarker for assessing the condition of patients with cerebral infarction (Lee et al., 2012). In this study, our main finding was that the level of serum 4-HNE in patients with recurrent cerebral infarction was significantly higher than that in patients with no-recurrent cerebral infarction. In addition, multivariate regression also confirmed that the high level of serum 4-HNE was an independent risk factor for the recurrence of cerebral infarction, and linear analysis confirmed that its level was positively correlated with serum triglyceride and LDL-C levels. These results indicated that serum 4-hydroxynonenal might increase the risk of recurrence in patients with primary cerebral infarction.

The pathological process of cerebral ischemia is extremely complex. After the ischemic injury, brain tissue will undergo changes, such as energy metabolism disorder, central neurotransmitter disorder, oxidative stress injury, and inflammatory response, leading to complex pathophysiological changes and apoptosis of neurons (Chamorro et al., 2016; Orellana-Urzúa et al., 2020). In recent years, the theory of oxidative stress has become a hotspot in ischemic stroke research. Hypoxia can cause tissues to produce large amounts of oxygen free radicals that act on unsaturated fatty acids in the cell membrane, causing peroxidation of membrane lipids, which in turn leads to cellular damage and the formation of lipid peroxides (Nathaniel et al., 2015; Jiang et al., 2020). 4-HNE is an aldehyde substance closely related to oxidative stress and lipid peroxidation, and oxidative stress is one of the main factors causing ischemic injury, suggesting that the increase of serum 4-HNE might be one of the main mechanisms underlying the increased risk of stroke induced by oxidative stress (Poli and Schaur, 2000; Liu et al., 2020). Importantly, a previous study found that serum 4-HNE level was significantly higher in patients with aspirin-resistant cerebral infarction than in patients with aspirin-sensitive cerebral infarction, and the recurrence rate of aspirin-resistant cerebral infarction patients was significantly higher than that of aspirin-sensitive patients with cerebral infarction, indirectly suggesting that serum 4-HNE may be associated with the recurrence of cerebral infarction (Guo et al., 2020).

On the one hand, as a consequence of cerebral ischemia, post-cerebral ischemia causes increased oxidative stress in the brain, ultimately leading to increased 4-HNE levels (McKracken et al., 2001; Guo et al., 2017). On the other hand, increased 4-HNE levels increase brain damage from ischemic stroke (Dou et al., 2012; Shoeb et al., 2014). To elaborate, former studies have revealed that excess 4-HNE may cause severe biotoxicity to cells through various pathways (Geib et al., 2021; Schröter et al., 2021), such as the induction of intramolecular and intermolecular cross-linking of proteins by 4-HNE and inhibition of protein synthesis by modifying the relevant sites of thiol-containing proteins. Moreover, 4-HNE binds to the sulfhydryl group of intracellular glutathione, which reduces the consumption of glutathione, weakens the intracellular antioxidant capacity, and further aggravates intracellular oxidative stress (Balogh and Atkins, 2011). Moreover, as the end product of lipid peroxidation, 4-HNE can induce the aggregation and activation of macrophages, induce the expression of inflammatory factors, promote the occurrence of inflammation, and aggravate cerebral ischemia injury (Liu et al., 2020; Hsu et al., 2022). As the severity of ischemic brain injury is closely related to the recurrence of cerebral infarction, the level of serum 4-HNE may be one of the main mechanisms related to the recurrence of cerebral infarction by affecting the progress of ischemic brain injury.

Another possible mechanism by which 4-HNE increases recurrence in patients with cerebral infarction is that 4-HNE affects platelet aggregation. Platelets is associated with the pathophysiology of stroke and their reactivity is not only an important clinical indicator of stroke (Järemo et al., 2013; Järemo et al., 2015), but also significantly correlated with the degree of brain injury and the risk of recurrence in patients with cerebral infarction (Guo et al., 2021; Yuan et al., 2021). Platelet-inhibiting drugs, such as aspirin, are effective in reducing the recurrence rate of cerebral infarction, while aspirin resistance is an independent risk factor for recurrence of cerebral infarction (Guo et al., 2020). Importantly, previous studies found that 4-HNE could affect platelet aggregation and might act as a negative feedback regulator of platelet function (Chapple et al., 2013; Ravi et al., 2016). In addition, we found that the high level of 4-HNE is more likely to play a negative feedback regulation role in platelet aggregation when LDL level is increased, suggesting that the influence mechanism of 4-HNE on the recurrence risk of patients with cerebral infarction may be similar to aspirin resistance (Malle et al., 1995).

## Conclusion

All in all, our results in the present study showed that serum 4-HNE was higher in patients with recurrent cerebral infarction, and serum 4-HNE was an independent risk factor for recurrence in patients with primary cerebral infarction.

## Data availability statement

The original contributions presented in the study are included in the article/supplementary material, further inquiries can be directed to the corresponding author.

## Ethics statement

This study was approved by the ethics committee of Hebei North University Hospital. The patients/participants provided their written informed consent to participate in this study.

## Author contributions

XL and ZL were mainly responsible for the writing and research design of the article. MB and LF were mainly responsible for data analysis. XL was responsible for ensuring that the descriptions are accurate and agreed by all authors. All authors contributed to the article and approved the submitted version.
